# Editorial

**DOI:** 10.1093/jrr/rrt215

**Published:** 2014-01

**Authors:** Kenshi Komatsu

The *Journal of Radiation Research* (JRR) has served as an official journal for the scientific communication of the Japan Radiation Research Society (JRRS) for more than 50 years. Partnered with the Japanese Society for Therapeutic Radiology and Oncology (JASTRO) since 2009, JRR has entered into a new phase of growth over multidisciplinary radiation research fields including biology, oncology, chemistry, physics, epidemiology and environmental sciences. Since 2012, JRR has shifted to a new partnership with Oxford University Press, which has the potential to be successful in the coming years. JRR ended 2013 with more than 340 new submissions compared to previous years.

The quality and impact of JRR are recognized throughout the multidisciplinary fields in radiation research. We have been providing an international forum for radiation scientists, but this trend has been changing in recent years in some statistics. JRR reached its highest impact factor of 2.034 in 2009, but it declined to 1.447 in 2012.

As I step in as a new Editor-in-Chief from 2014, I would firstly like to aim to further international influence. The continuous high rate of papers from outside of Japan is indeed pleasure for us, but we would also like to pursue increased number of high quality papers. In order to achieve that, we hope to keep working on smoother review process to make prompt decisions to benefit authors and to keep up with the international standard for this discipline.

Finally, I would like to thank previous EIC, Yoshiya Furusawa, who has contributed immensely for the development of the journal. I would hereby like to express my gratitude to all personnel for their continuous support and would like to ask for your further cooperation to improve the quality and expand the international prominence of the *Journal of Radiation Research*.

